# Turnover intention and its related factors of clinical research coordinator in Hunan, China: a cross-sectional study

**DOI:** 10.1038/s41598-024-66960-8

**Published:** 2024-07-11

**Authors:** Juan Li, JinHua Li, ZhengDi She, LiWen Guo, ShanZhi Gu, Wen Lu

**Affiliations:** Hunan Cancer Hospital, Changsha, Hunan China

**Keywords:** Health occupations, Oncology

## Abstract

To explore the related factors of turnover intention in clinical research coordinators (CRCs) and assess the mediating effects of professional identity on the association between job burnout and turnover intention. In China, CRC has become increasingly common among clinical trial teams in recent years. However, limited published research focused on the status of turnover intention in CRCs. We invited all the 220 CRCs currently working at Hunan Cancer Hospital located in Changsha city in the central south of China from March to June 2018. Participants were asked to complete structured questionnaires regarding basic demographic information, job burnout, professional identity and turnover intention. A total of 202 participants were included in this study, with a response rate of 91.82%. The main reason for turnover intention among CRCs was human resources, followed by communications, management and material resources (per item score in each dimension: 2.14 vs. 2.43 vs. 2.65 vs. 2.83). All the correlations among job burnout, professional identity and turnover intention were statistically significant, with coefficients ranging from −0.197 to 0.615. Multiple liner regression analysis showed that older age, longer workhours per week, and lower level of professional identity were associated with the prevalence of turnover intention among CRCs. Besides, the association between job burnout and turnover intention was fully mediated by professional identity. This study revealed the status and causes of turnover intention among Chinese CRCs. Effective measures on decreasing working time and improving professional identity should be taken in order to reduce CRCs’ turnover intention.

## Introduction

In recent years, the number of clinical trials in China has rapidly increased to meet the needs of development of new drugs. Until September 2021, 14,615 applications of clinical trials were registered by the Center for Drug Evaluation of National Medical Products Administration (NMPA) in China^1^. We are facing a crucial problem—how to ensure the authenticity and reliability of such huge clinical trial data. In fact, NMPA issued The Announcement of Self-examination and Inspection of Drug Clinical Trial Data in July 2015, launching the most stringent drug registration self-examination and inspection in history^2^. Every drug registration application must go through an onsite inspection of clinical trial to prove that its data are authentic before it can be approved.In order to conduct high-quality clinical trials, clinical research coordinator (CRC) is necessary to assist investigators with simple but tedious tasks^3^. Generally speaking, an eligible CRC usually has an educational background in medicine, nursing or pharmacy, and is directly employed in medical institutions^4^. As the person who interacts most with the subjects^5^, CRC is able to coordinate a variety of activities and improve the compliance of subjects with protocols^6^.

The need for CRCs is still expanding worldwide. For example, the job market for CRCs in the U.S. is estimated to grow by 9.9% between 2016 and 2026^7^. While it brings opportunities, it also ushers in more challenges. The strengthened regulation of clinical trials has raised the expectations for CRCs, calling for a greater emphasis on skills, training, and medical knowledge^8^. To ensure that the hired CRCs carry out their job properly and responsibly, companies invest a great deal of time and training, making it costly once CRCs leave their current job^9^. However, the turnover of CRCs is increasingly frequent in few decades. The CRC role has now become a temporary position for coordinators seeking to gain clinical experience prior to medical school or other graduate positions^10^. A study conducted in Italy discovered that only 13.8% of the CRCs was hired with a permanent contract, which would directly affect their future career plans^11^. China also faces the problem of excessive turnover of clinical research coordinator^12^. Therefore, reducing the turnover rates of CRCs is urgently needed for clinical research. Turnover intention is considered to be the main and immediate precursor of actual turnover behavior^13,14^.

However, the emerged abundant researches on CRCs in recent years, mostly concentrated on the application and impact of CRCs in clinical trials^15–17^. Little study focused on CRCs’ personal psychological characteristics including turnover intention, job burnout, or professional identity.To our knowledge, only one study has deeply investigated the severe situation of turnover among CRCs^18^. Focusing on CRCs’ personal psychological characteristics can help organizations and researchers identify areas for intervention, develop targeted support systems, and improve recruitment and selection strategies, ultimately leading to higher job satisfaction, lower burnout, and reduced turnover among CRCs.

The Job Embeddedness theory suggests that employees’ turnover intention may depend on the sense of embeddedness towards their professional and social environment^19^. However, this sense of professional identity can be gradually eroded by job burnout, which may ultimately increase employees’ likelihood of leaving their job^20^. Therefore, we hypothesized that professional identity directly affects turnover intention, and mediates the association between job burnout and turnover intention.In this study, we aimed to explore: (1) the current situation and influencing factors of turnover intention in CRCs; and (2) the role of professional identity between the association of job burnout with turnover intention. The findings may provide government and medical institutions with strategies to manage turnover intention among CRCs.

## Methods

### Study population

A cross-sectional survey was conducted in Hunan Provincial Tumor Hospital in China from 1st March 2021 to 30th June 2021. All methods were performed in accordance with the ethical principles of the Declaration of Helsinki.Targeted participants included CRCs who were currently employed in the hospital for at least one year. Participation in the survey was voluntary and we invited 220 CRCs to take part in this study. The inclusion and exclusion criteria are as follows: (1) The inclusion criteria: all the CRCs who were currently employed in the hospital for at least one year, regardless of their intention to leave the hospital in the future. (2) The exclusion criteria: CRCs who rejected to participant in this study. Eventually, a total of 202 participants were recruited for this study, with a response rate of 91.82%. The local institutional review board approved this study. All the participating CRCs gave their informed consent at his or her enrollment.

### Instrument and measurement

#### Demographic characteristics

We collected participants’ information on demographic characteristics including age, gender, educational level (associate’s degree or below, and bachelor’s degree or above), marital status (married, unmarried, and other), per capita monthly income (< 6000, 6000–8000, 8000–10,000, and > 10,000 Chinese Yuan (CNY)), working years (1–2, 3–4, ≥ 5 years), working hours per week (≤ 40 h, 40–50 h, 50–60 h, > 60 h), number of patients to manage (1–10, 11–20, 21–30, > 30), number of children to raise (0, 1, ≥ 2), and level of CRC (CRC, Senior CRC, and CRC leader).

#### Turnover intention

Turnover intention was assessed by the adapted Chinese version of the MISSCARE survey scale. The original scale was invented by Kalisch & Williams in 2009 ^21^, and the Chinese version was developed by Si and Qian^22^. Both scales consisted of two parts that part one evaluated the degree of missed nursing care and part two addressed the reasons^21,22^. Different from the original version whose part two consisted of three dimensions with 17 items, the Chinese version included 19 items in part two, indicating four factors named as management, communications, human resources and material resources^22^. In this study, we utilized the part two of Chinese version and adapted some of the descriptions to make them more appropriate to CRCs. Higher scores of the scale indicate lower level of turnover intention among CRCs.The adapted survey scales for CRCs were validated by confirmatory factor analysis (data not shown).

#### Job burnout

Job burnout was measured using the 22-item Maslach Burnout Inventory-Human Services Survey (MBI-HSS), with each item ranging from ‘never’ (0 point) to ‘every day’ (6 points)^23^. This scale contains three dimensions, including emotional exhaustion (9 items), depersonalization (5 items), and personal accomplishment (8 items). Higher scores on emotional exhaustion and depersonalization indicate higher level of job burnout, while the score of personal accomplishment is inversely correlated with job burnout. The Chinese version of MBI-HSS has been validated and widely used in Chinese population^24–26^.

#### Professional identity

Professional identity was assessed by Professional Identity Scale for Nurses developed by Liu and colleagues^27^. This 30-item instrument is a 5-point Likert scale comprising 5 dimensions: professional identity evaluation (9 items), professional social support (6 items), professional social proficiency (6 items), dealing with professional frustration (6 items), and professional self-reflection (3 items). The total score ranges from 30 to 150 points, with higher score indicating a higher level of professional identity. The scale showed good reliability with a Cronbach's coefficient of 0.94 and a split-half reliability of 0.88 in Chinese nurses^27^. In order to adapt the scale to CRCs, we modified some elements of the scale as appropriate.

### Statistical analysis

Descriptive statistics were presented as mean with standard deviation (SD). Student t test or one-way analysis of variance test was utilized to examine the difference of turnover intention scores among subgroups of individual demographic variables, as appropriate. Pearson correlation analyses were applied to determine the relationships among turnover intention, job burnout and professional identity. In addition, multiple linear analysis was used to explore the potential related factors of turnover intention.Before each linear analysis, the four assumptions including linear, independence, normality and homoscedasticity were checked.

Previous studies have revealed that job burnout and professional identity were potential predictors of turnover intention^28,29^. In this study, we hypothesized that job burnout may lead to turnover intention among CRCs through lack of professional identity. Thus, mediation analysis was performed to address whether professional identity mediated the association between job burnout and turnover intention in CRCs. We used the PROCESS for SPSS with 5000 bootstrap resamples^30^. Whether the mediating effect existed depended on the significance of indirect effect between job burnout and turnover intention. In brief, full mediation was defined when indirect effect was significant whereas direct effect was non-significant, and partial mediation was defined when both indirect and direct effect were significant.

All statistical analyses were performed using the SPSS 21.0 software package (SPSS Institute, Chicago) with two-tailed tests where *P* < 0.05 was considered statistically significant.

### Ethics approval

This study was reviewed and approved by the Ethics Committee for Clinical Trials, Hunan cancer hospital, and written informed consent was obtained for all participants. All participants enrolled in the study provided written informed consent.

## Results

### Characteristics of study population

Table 1 summarizes the demographics of the study participants and the distribution of turnover intention scores in categorical items. The sample comprised 202 CRCs, 98.0% of whom were females and whose ages ranged from 16 to 35 years (Mean ± SD: 25.37 ± 3.04). Age groups showed differences in the mean scores of management dimension (*P* = 0.015). As for working hours, 6.9%, 53.5%, 28.7% and 10.9% of CRCs worked for less than 40 h, 40 to 50 h, 50 to 60 h and more than 60 h per week, respectively. Groups for working hours per week performed differences in the mean scores of management dimension (*P* = 0.038), communication dimension (*P* = 0.005), human resources dimension (*P* < 0.001) and the total score (*P* = 0.003). Of the participants, the majority (85.6%) had no child yet and 11.9% CRCs had only one child to raise. Groups based on number of children to raise showed differences in the mean scores of management dimension (*P* = 0.043). In addition, the turnover intention score had no statistical difference in the other characteristics of study population (all *P* > 0.05).

**Table 1:** Characteristics of CRCs and differences in turnover intention among different characteristics groups (mean ± SD).

Variables	N (%)	Management	Communication	Human resources	Material resources	Total score
Total	202	15.88 ± 4.77	14.55 ± 4.43	8.55 ± 2.62	8.48 ± 2.57	47.76 ± 12.31
Gender						
Men	4 (2.0)	16.00 ± 5.48	15.25 ± 4.57	7.50 ± 1.73	8.50 ± 3.00	47.25 ± 13.30
Women	198 (98.0)	15.87 ± 4.77	14.54 ± 4.44	8.57 ± 2.63	8.47 ± 2.57	47.46 ± 12.33
t		0.052	0.316	−0.808	0.019	−0.034
P		0.958	0.752	0.420	0.985	0.973
Age (years)						
≤ 25	120 (59.4)	15.88 ± 4.80	14.63 ± 4.59	8.75 ± 2.74	8.37 ± 2.51	47.63 ± 12.58
26–30	67 (33.2)	16.60 ± 4.48	14.85 ± 4.15	8.37 ± 2.42	8.85 ± 2.58	48.67 ± 11.36
> 30	15 (7.4)	12.67 ± 4.76	12.60 ± 4.19	7.73 ± 2.43	7.67 ± 2.85	40.67 ± 12.83
F		4.298	1.636	1.234	1.577	2.662
P		0.015	0.197	0.293	0.209	0.072
Educational level						
Associate’s degree or below	64 (31.7)	15.95 ± 4.53	14.72 ± 4.35	8.77 ± 2.50	8.09 ± 2.51	47.53 ± 12.06
Bachelor’s degree or above	138 (68.3)	15.84 ± 4.89	14.48 ± 4.49	8.45 ± 2.68	8.65 ± 2.58	47.42 ± 12.47
T		0.156	0.358	0.798	−1.442	0.059
P		0.876	0.721	0.426	0.151	0.953
Marital status						
Unmarried	157 (77.7)	15.99 ± 4.78	14.68 ± 4.45	8.57 ± 2.63	8.47 ± 2.49	47.70 ± 12.31
Married	44 (21.8)	15.57 ± 4.80	14.11 ± 4.67	8.52 ± 2.64	8.41 ± 2.82	46.61 ± 12.56
Others	1 (0.5)	12.00	15.00	7.00	12.00	46.00
F		0.462	0.279	0.179	0.957	0.140
P		0.631	0.757	0.836	0.386	0.870
Income (RMB/month)						
< 6000	87 (43.1)	15.97 ± 4.87	14.43 ± 4.73	8.72 ± 2.70	8.29 ± 2.58	47.40 ± 13.01
6000–8000	67 (33.2)	15.91 ± 4.69	14.72 ± 4.28	8.39 ± 2.55	8.66 ± 2.61	47.67 ± 11.74
8000–10,000	35 (17.3)	16.31 ± 4.73	15.00 ± 4.25	8.63 ± 2.69	8.51 ± 2.51	48.46 ± 11.91
> 10,000	13 (6.4)	13.92 ± 4.68	13.38 ± 3.82	8.00 ± 2.45	8.69 ± 2.59	44.00 ± 12.20
F		0.834	0.470	0.411	0.298	0.422
P		0.476	0.703	0.745	0.827	0.737
Working years (years)						
1–2	110 (54.5)	15.75 ± 4.69	14.32 ± 4.49	8.67 ± 2.60	8.37 ± 2.46	47.12 ± 12.15
3–4	59 (29.2)	16.17 ± 4.35	15.10 ± 4.19	8.42 ± 2.63	8.76 ± 2.51	48.46 ± 11.29
≥ 5	33 (16.3)	15.76 ± 5.77	14.36 ± 4.72	8.36 ± 2.75	8.30 ± 3.03	46.79 ± 14.68
F		0.156	0.634	0.271	0.529	0.283
P		0.855	0.532	0.763	0.590	0.754
Working hours (hours/week)						
< 40 h	14 (6.9)	16.36 ± 6.17	16.21 ± 5.58	9.71 ± 3.47	8.79 ± 3.17	51.07 ± 17.31
40–50 h	108 (53.5)	16.64 ± 4.87	15.21 ± 4.40	8.99 ± 2.53	8.73 ± 2.41	49.57 ± 12.06
50–60 h	58 (28.7)	15.10 ± 4.14	13.88 ± 4.00	8.16 ± 2.35	8.28 ± 2.38	45.41 ± 10.51
> 60 h	22 (10.9)	13.86 ± 4.21	12.05 ± 3.89	6.68 ± 2.15	7.55 ± 3.22	40.14 ± 11.21
F		2.859	4.463	6.619	1.517	4.857
P		0.038	0.005	< 0.001	0.211	0.003
Number of patients under manage						
1–10	113 (55.9)	15.96 ± 4.73	14.67 ± 4.54	8.83 ± 2.77	8.32 ± 2.49	47.79 ± 12.41
11–20	58 (28.7)	16.02 ± 4.59	14.69 ± 4.38	8.24 ± 2.43	8.83 ± 2.80	47.78 ± 12.39
21–30	10 (5.0)	16.10 ± 5.49	15.50 ± 4.33	8.80 ± 1.81	8.50 ± 2.42	48.90 ± 12.64
> 30	21 (10.4)	14.90 ± 5.35	13.10 ± 4.01	7.76 ± 2.49	8.33 ± 2.48	44.10 ± 11.74
F		0.324	0.954	1.375	0.523	0.604
P		0.808	0.416	0.252	0.667	0.613
Number of children under raise						
0	173 (85.6)	16.01 ± 4.67	14.61 ± 4.44	8.59 ± 2.60	8.50 ± 2.50	47.71 ± 12.06
1	24 (11.9)	16.00 ± 5.15	14.88 ± 4.54	8.63 ± 2.84	8.58 ± 2.87	48.08 ± 13.54
≥ 2	5 (2.5)	10.60 ± 4.22	11.20 ± 2.95	6.80 ± 1.79	7.00 ± 3.54	35.60 ± 11.06
F		3.206	1.513	1.146	0.856	2.421
P		0.043	0.223	0.320	0.426	0.091
Level of CRC						
CRC	120 (59.4)	16.04 ± 4.91	14.60 ± 4.61	8.67 ± 2.67	8.32 ± 2.57	47.63 ± 12.77
Senior CRC	52 (25.7)	16.58 ± 4.39	15.13 ± 4.24	8.44 ± 2.62	9.17 ± 2.49	49.33 ± 11.09
CRC leader	30 (14.9)	14.00 ± 4.50	13.37 ± 3.93	8.27 ± 2.46	7.90 ± 2.52	43.53 ± 11.96
F		3.014	1.536	0.336	2.961	2.159
P		0.051	0.218	0.715	0.054	0.118

Student *t* test (*t* value) or one-way analysis of variance test (F value) was utilized, as appropriate.

### Reasons for turnover intention

Table 2 presents total means of each dimension of reasons for turnover intention in CRCs. By examining the total mean score for each dimension, human resources had the lowest mean score (Mean ± SD: 2.14 ± 0.66), and thus were the most prevalent reason for turnover intention; while the following mean scores for communications, management and material resources dimensions were 2.43 (SD = 0.74), 2.65 (SD = 0.79) and 2.83 (SD = 0.86), respectively.

**Table 2:** Mean score per item for dimensions of reasons for turnover intention among CRCs.

Dimensions of reasons for turnover intention	Mean	SD
Mean score per item for management reasons	2.65	0.79
Mean score per item for communication reasons	2.43	0.74
Mean score per item for human resources reasons	2.14	0.66
Mean score per item for material resources reasons	2.83	0.86

*SD* standard deviation.

### The correlation among job burnout, professional identity, and turnover intention

Pearson correlation analysis between professional identity and turnover intention revealed a positive correlation (r = 0.413, *P* < 0.001) (see Table 3), which indicated that higher level of professional identity was related to lower level of turnover intention among CRCs. As for each job burnout dimension, higher levels of motional exhaustion (r = −0.197, *P* < 0.01), depersonalization (r = −0.212, *P* < 0.01) and felling of low personal accomplishment (r = 0.198, *P* < 0.01) were related to higher level of turnover intension. In addition, each job burnout dimension also displayed statistical correlations with professional identity (motional exhaustion: r = −0.365; depersonalization: r = −0.291; felling of low personal accomplishment: r = 0.364). In sum, job burnout, professional identity and turnover intention were correlated with each other.

**Table 3:** Descriptive statistics and correlations between variables.

Variable	Mean ± SD	1	Job burnout			5
			2	3	4	
1. Professional identity	98.85 ± 17.39	–				
Job burnout						
2. Motional exhaustion	24.91 ± 13.59	−0.365***	–			
3. Depersonalization	7.50 ± 6.38	−0.291***	0.615***	–		
4. Felling of low personal accomplishment	26.35 ± 9.98	0.364***	0.126*	0.117*	–	
5. Turnover intention	47.46 ± 12.31	0.413***	−0.197**	–0.212**	0.198**	–

*SD* standard deviation; *p < 0.05, **p < 0.01, ***p < 0.001.

Pearson correlation analyses were applied to calculate the correlations among turnover intention, job burnout and professional identity.

### The influencing factors of turnover intention assessed by multiple linear regression analysis

The factors associated with CRCs’ turnover intention are presented in Table 4. All the assumptions of linear regression including linear, independence, normality and homoscedasticity were met (data not show).From Tables 1 and 3, we firstly extracted the factors that were statistically associated or correlated with turnover intention. Then, those factors were included in the multiple liner regression model. According to the regression analysis, CRCs who were relatively older, who worked longer per week were more likely to report a higher risk of turnover intention. While, higher level of professional identity was independently associated with lower risk of turnover intention among CRCs.

**Table 4:** Multiple linear analysis for exploring the potential related factors of turnover intention.

Variables	β	SE	95% CI	Wald χ^2^	*P*
Age					
≤ 25	0				
26–30	−0.889	1.7718	−4.362, 2.583	0.252	0.616
> 30	−10.283	3.2954	−16.741, −3.824	9.736	0.002
Working hours per week					
< 40 h	0				
40–50 h	−3.978	3.0850	−10.024, 2.069	1.662	0.197
50–60 h	−7.201	3.2833	−13.636, −0.766	4.810	0.028
> 60 h	−11.537	3.8624	−19.107, −3.967	8.922	0.003
Number of children					
0	0				
1	3.438	2.5838	−1.626, 8.502	1.770	0.183
≥ 2	−2.627	5.1436	−12.712, 7.459	0.261	0.610
Job burnout					
Motional exhaustion	0.032	0.0638	−0.093, 0.157	0.255	0.614
Depersonalization	−0.103	0.1286	−0.355, 0.149	0.642	0.423
Reduced personal accomplishment	0.158	0.0818	−0.003, 0.318	3.709	0.054
Professional identity	0.246	0.0528	0.143, 0.350	21.771	< 0.001

*SE* standard error, *CI* confidence interval.

### Mediation effect of professional identity on job burnout and turnover intention

**Figure 1:**
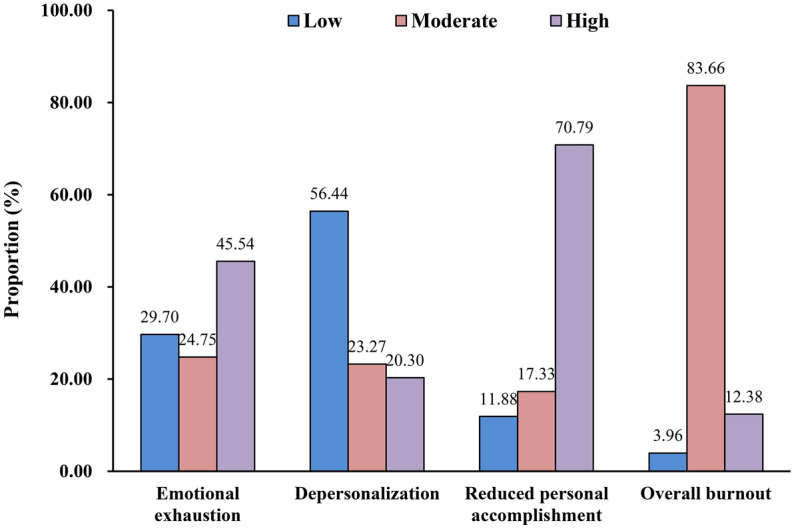
The constructed mediation models of professional identity on the association between job burnout [motional exhaustion (**a**), depersonalization (**b**), felling of low personal accomplishment (**c**)] and turnover intention.

Figure 1 illustrates the constructed mediation model of professional identity on job burnout and turnover intention. For motional exhaustion subscale, the standardized effect value of motional exhaustion on professional identity was −0.4664 (path a1, *P* < 0.001) and the standardized effect value of professional identity on turnover intention was 0.2787 (path b1, *P* < 0.001). Thus, the standardized indirect effect value of motional exhaustion on turnover intention through professional identity was -0.1300 (path a1*b1, *P* < 0.001), confirming a significant mediation effect. However, the direct effect of motional exhaustion on turnover intention was not statistically significant (path c’ = −0.0486, *P* = 0.4391). Therefore, professional identity fully mediated the association between motional exhaustion and turnover intention. In the same manner, professional identity also fully mediated the association between depersonalization and turnover intention, and the association between reduced personal accomplishment and turnover intention. More detailed information on the outputs of mediation analysis was shown in Table 5.

**Table 5:** Mediation modeling results assessing the effect of professional identity on the relationship between job burnout and turnover intention.

Predictor	Effect	Path	R^2^	β	SE	t	LLCI	ULCI	*P*-value
Motional exhaustion	Total effect	Motional exhaustion → Turnover intention (path c1)	0.0389	−0.1786	0.0628	−2.8445	−0.3024	−0.0548	0.0049
	Direct effect	Motional exhaustion → Professional identity (path a1)	0.1330	−0.4664	0.0842	−5.5392	−0.6325	−0.3004	< 0.001
		Professional identity → Turnover intention (path b1)	0.1731	0.2787	0.0490	5.6845	0.1820	0.3753	< 0.001
		Motional exhaustion → Turnover intention (path c1’)		−0.0486	0.0627	−0.7753	−0.1722	0.0750	0.4391
	Indirect effect	Motional exhaustion → Professional identity → Turnover intention (path a1*b1)	−	−0.1300	0.0382	−	−0.2195	−0.0668	< 0.001
Depersonalization	Total effect	Depersonalization → Turnover intention (path c2)	0.0451	−0.4100	0.1333	−3.0751	−0.6729	−0.1471	0.0024
	Direct effect	Depersonalization → Professional identity (path a2)	0.0847	−0.7929	0.1843	−4.3014	−1.1564	−0.4294	< 0.001
		Professional identity → Turnover intention (path b2)	0.1800	0.2717	0.0475	5.7195	0.1781	0.3654	< 0.001
		Depersonalization → Turnover intention (path c2’)		−0.1945	0.1295	−1.5023	−0.4498	0.0608	0.1346
	Indirect effect	Depersonalization → Professional identity → Turnover intention (path a2*b2)	–	−0.2155	0.0689	−	−0.3768	−0.0998	< 0.001
Reduced personal accomplishment	Total effect	Reduced personal accomplishment → Turnover intention (path c3)	0.0393	0.2445	0.0855	2.8611	0.0760	0.4130	0.0047
	Direct effect	Reduced personal accomplishment → Professional identity (path a3)	0.1323	0.6334	0.1147	5.5220	0.4072	0.8596	< 0.001
		Professional identity → Turnover intention (path b3)	0.1733	0.2783	0.0490	5.6793	0.1816	0.3749	< 0.001
		Reduced personal accomplishment → Turnover intention (path c3’)		0.0683	0.0853	0.8002	−0.1000	0.2365	0.4246
	Indirect effect	Reduced personal accomplishment → Professional identity → Turnover intention (path a3*b3)	–	0.1762	0.0557	–	0.0849	0.3071	< 0.001

## Discussion

### Key findings

To our knowledge, this may be the first study focusing on the turnover intention among CRCs in China. In this study, three major findings were obtained. Firstly, the main reason for turnover intention among CRCs was human resources, followed by communications, management and material resources. Secondly, older age, longer workhour per week, and lower level of professional identity were associated with the prevalence of turnover intention among CRCs. Thirdly, professional identity fully mediated the association between job burnout and turnover intention. Our research findings may not only expand the understandings of turnover intention in CRCs, but also provide evidence for policy-making to reduce turnover intention of CRCs in China.

### Epidemiological evidence

Vanderbilt University Medical Center conducted a survey on CRCs over a 12-month period from October 2017 to September 2018^18^. The study discovered 9 significant predictors related to retention including salary, level of CRC and so on. Nevertheless, in our study, salary and level of CRC were not significantly associated with turnover in CRCs. Instead, we found older age, longer working hours per week, and lower level of professional identity were predictors of turnover intention. One possible reason was the different occupational environment between the USA and China. CRCs in China may care less about their own career development under China's immature CRC management system. On the other hand, the American study did not collect information related to age, level of education, or working hours, which were taken into account in our study. In fact, these basis characteristics have been suggested influencing factors with turnover of other medical staff, such as nurses and doctors^31–33^.

In this study, we applied adapted MISSCARE survey scale to evaluate the turnover intention of CRCs^22^. The scale consisted of four factors named as management, communications, human resources and material resources. According to our results, human resources were the most prevalent reason for turnover intention of CRCs. Such findings were consistent with most studies on nurses^34,35^. In fact, CRCs and nurses were similar to some extent, at least their most time was spent on dealing with patients. Besides, they both may be faced with staffing inadequacy or heavy workloads, which belong to the human resources dimension. Such reasons could be effectively resolved by hospitals or companies through promotions on supportive work environment, work schedule management, and enhancing CRCs’ teamwork. In addition, CRCs also needed to improve their time management skills and working competence in order to deal with the tedious work.

### Mediation effect

In sociology, job burnout, professional identity and turnover intention have been researched extensively by sociologists. Many previous studies have revealed the complex relationships among the three variables in other occupational populations^28,29^. For example, Zhang et al.^28^ found that professional identity had an indirect negative effect on turnover intention through the mediating effect of burnout among general practitioners. In this study, we discovered that professional identity fully mediated the associations between job burnout and turnover intention among CRCs. The results seemed to be inconsistent with previous studies, because the mediator has changed from job burnout to professional identity. The phenomenon could be interpretated appropriately. For nurses or doctors, they were cultivated their sense of professional identity as early as their school years. Once they had a high degree of identity with their career, they would reduce the incidence of job burnout^36^, which in turn caused a reduction of turnover intention^37,38^. As for CRCs, they usually lack education or training on identity with their career when they were in school or pre-employment due to the undeveloped CRC management system in China. Thus, their unstable professional identity may be easily altered by feelings of burnout.

In our study, we found that professional identity had direct effect on turnover intention, which was consistent with previous studies^39,40^.When CRCs had a high level of professional identity with their career, they would be willing to devote more time and efforts to their work, without considering leaving their present career. This finding suggests that improving CRCs' professional identity could serve as an effective way to reduce turnover intention. Actions such as providing pre-job training, reducing the work intensity, extending break time, increasing communications between CRCs and principal investigators are warranted to improve the degree of professional identity of CRCs.

### Limitations

To our knowledge, to date, this is the first study to assess the situation of turnover intention among CRCs and to explore the role of professional identity in the association of job burnout with turnover intention in China. However, this study also has several limitations. First, our study could not confirm the temporality and causality of the observed relationships due to its cross-sectional design. Second, CRCs’ turnover intention and professional identity were evaluated by adapted MISSCARE survey scale and Professional Identity Scale^22,27^, respectively, whereas the two scales were initially developed for nurses. Worth mentioning, we were in the process of verifying the reliability and validity of the two adapted scales. Third, some potential influencing factors were not included into our questionnaire, such as work schedules, sleep quality, social support, health status, mental health and so on. Fourth, the study was conducted in a relatively narrow time frame from 1st March 2021 to 30th June 2021, which may result in seasonal bias. In addition, The R^2^ in the regression models showed a little small which may suggest a poor fit of the model to the data. However, it could still provide evidence of the mediating effect to some extent, even if the overall explanatory power of the model is limited. Finally, CRCs in our study were recruited from Hunan Cancer Hospital, which may limit the externality of our results to general hospitals or other specialized hospitals. Further research on this topic is needed to include more potential influencing factors and expand the sample selection in the future.

### Implications and recommendations for practice

CRCs play important roles in promoting project implementation and enhancing quality of clinical trials. Awareness and management of turnover intention in CRCs may stabilize the workforce in the field of clinical trials. Therefore, the government and medical institutions should establish a more developed management system to reduce the loss of CRCs. Measures such as enhancing CRCs’ professional identity and reducing working hours appropriately would be effective approaches.

## Conclusions

Our study may be the first to assess the situation of turnover intention among CRCs in China. It revealed that the main reason for turnover intention of CRCs was human resources. Age, working hours per week, and professional identity were influencing factors of turnover intention. Besides, professional identity had fully mediating effect on the association of job burnout with turnover intention. Institutional and policy changes such as reducing burdensome working hours and increasing professional identity should be implemented to lower turnover intentions among CRCs. A reasonable management system on CRCs is appealed in the near future.

## Data Availability

The datasets used and/or analyzed during the current study are available from the corresponding author on reasonable request.

## Abbreviations

NMPA: National Medical Products Administration
CRC: Clinical research coordinator
RMB: Renminbi
MBI-HSS: Maslach Burnout Inventory-Human Services Survey
SD: Standard deviation
